# Interaction of poly(ethylene glycol)-conjugated phospholipids with supported lipid membranes and their influence on protein adsorption

**DOI:** 10.1080/14686996.2016.1240006

**Published:** 2016-10-18

**Authors:** Toshihiro Yamamoto, Yuji Teramura, Toru Itagaki, Yusuke Arima, Hiroo Iwata

**Affiliations:** ^a^ Department of Reparative Materials, Institute for Frontier Medical Sciences, Kyoto University, Kyoto, Japan; ^b^ Department of Bioengineering, The University of Tokyo, Tokyo, Japan; ^c^ Department of Material Chemistry, Graduate School of Engineering, Kyoto University, Kyoto, Japan

**Keywords:** Poly(ethylene glycol)-conjugated phospholipid (PEG-lipids), supported lipid membrane, surface modification, membrane fluidity, hydrophobic interaction, 30 Bio-inspired and biomedical materials, 212 Surface and interfaces

## Abstract

We studied real-time interaction between poly(ethylene glycol)-conjugated phospholipids (PEG-lipids) and a supported lipid membrane by surface plasmon resonance (SPR) spectroscopy to understand dynamic behaviors of PEG-lipids on living cell membranes. Supported lipid membranes formed on a hydrophobic surface were employed as a model of living cell membrane. We prepared three kinds of PEG-lipids that carried alkyl chains of different lengths for SPR measurements and also performed fluorescence recovery after photobleaching (FRAP) to study the influence of acyl chain length on dynamics on the supported membrane. PEG-lipids were uniformly anchored to lipid membranes with high fluidity without clustering. Incorporation and dissociation rates of PEG-lipids into supported membranes strongly depended on the length of acyl chains; longer acyl chains reduced the incorporation rate and the dissociation rate of PEG-lipid. Furthermore, protein adsorption experiment with bovine serum albumin indicated that PEG modification prevented the adsorption of bovine serum albumin on such supported membrane.

## Graphical abstract

**Figure:**
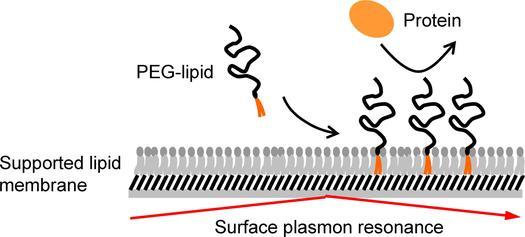

## 1. Introduction

Various synthetic and natural polymers have been widely used to modify the surface of materials to modulate the immunogenicity after implantation or infusion into the human body.[1–3] Recently, the surfaces of cells have been engineered in order to improve graft survival in cell transplantation.[4,5] Approaches for modifying cell surfaces include: covalent conjugation to membrane proteins,[6–11] electrostatic interaction with a negatively charged cell surface,[12–14] and hydrophobic interaction with a lipid bilayer of a cell.[15,16] Cell surface modification with polymers should be both highly efficient and minimally cytotoxic. In addition, modified polymers on cell surfaces are expected to suppress interaction with proteins and cells, which might lead to undesired biological responses.[17]

We have studied amphiphilic polymers including poly(ethylene glycol)-phospholipid conjugate (PEG-lipid) derivatives and partially alkylated poly(vinyl alcohol)s (PVA-alkyls) for cell surface modifications.[16,18] The alkyl chains of these polymers are inserted in the lipid bilayer of living cells through hydrophobic interaction, resulting in anchoring of the polymers on cell surface. Furthermore, biomolecules could be immobilized to anchored polymers on the cell surface for enhancing graft survival.[19] Anchored polymers, however, gradually disappeared from the cell surface.[16,18,20] The stability and retention of amphiphilic polymers on a living cell surface must be enhanced to effectively modify the cell surface. However, it is difficult to determine which pathways are involved in the disappearance of those polymers, by biological exclusion and/or by a physicochemical route such as spontaneous dissociation from the surface of living cells.

In this study, we investigated interaction between PEG-lipids and a model cell membrane. Supported lipid membrane, which is lipid layer formed on a solid support, was used as a mimic of the living cell membrane, although there are some differences such as membrane proteins and surface charge. We examined interaction between supported lipid membrane and PEG-lipids with different alkyl chains using surface plasmon resonance (SPR) and lateral fluidity of PEG-lipids using fluorescence recovery after photobleaching (FRAP). Furthermore, interaction with serum albumin was examined to study protein adsorption on PEG-lipid modified membranes.

## 2. Materials and methods

### 2.1. Materials

α-*N*-Hydroxysuccinimidyl-ω-Boc protected-amino-PEG (NHS-PEG-NH_2_-Boc, Mw: 5000), 1,2-dimyristoyl-*sn*-glycerol-3-phosphatidylethanolamine (DMPE), 1,2-dipalmitoyl-*sn*-glycerol-3-phosphatidylethanolamine (DPPE), and 1,2-distearoyl-*sn*-glycerol-3-phosphatidylethanolamine (DSPE) were purchased from NOF Corporation (Tokyo, Japan). Dichloromethane, chloroform, toluene, diethyl ether, and *n*-butyl amine were obtained from Nacalai Tesque (Kyoto, Japan). Methoxyl-PEG-succinimidyl propionate (MeO-PEG-NHS, Mw 5000) was purchased from Nektar Therapeutics (San Carlos, CA, USA). L-α-Phosphatidylcholine from egg yolk (EggPC) and bovine serum albumin (BSA) were purchased from Sigma-Aldrich Co. (St Louis, MO, USA). Fluorescein isothiocyanate (FITC) was obtained from Dojindo Laboratories (Kumamoto, Japan). Phosphate-buffered saline (PBS) was obtained from Nissui Pharmaceutical, Co., Ltd (Tokyo, Japan). 1-Hexadecanethiol was purchased from Tokyo Chemical Industry Co., Ltd (Tokyo, Japan). Trifluoroacetic acid (TFA) and phospholipid C-test wako were purchased from Wako Pure Chemical Industries, Ltd (Osaka, Japan). Octadecyl triethoxysilane was supplied from Shin-Etsu Chemical Co., Ltd (Tokyo, Japan). L-α-Palmitoyl-NBD dodecanoyl phosphatidylethanolamine was purchased from Avanti Polar Lipids, Inc. (Alabaster, AL, USA).

### 2.2. Synthesis of PEG-conjugated phospholipid (PEG-lipid) and FITC-labeling

MeO-PEG-DPPE was synthesized by mixing MeO-PEG-NHS (170 mg) and DPPE (20 mg) in dichloromethane (5 ml) for three days at room temperature (RT). The product was precipitated with diethyl ether, extracted into chloroform, and then freeze-dried. Synthesis of PEG-lipids was confirmed by ^1^H-NMR.[18]

NH_2_-PEG-lipids were synthesized from NHS-PEG-NH_2_-Boc and DMPE or DPPE or DSPE, respectively, and were then labeled with FITC, as reported previously.[15,16] FITC–PEG-lipid was dissolved in PBS, followed by purification by gel permeation chromatography (Sephadex G-25; GE Healthcare Bio-Sciences, Uppsala, Sweden). Synthesis of PEG-lipids and subsequent conjugation with FITC were confirmed by proton nuclear magnetic resonance (^1^H-NMR).[18,20]

### 2.3. Preparation of alkanethiol monolayer surfaces

Glass plates (BK7, 25 × 25 × 1 mm, Arteglass Associates Co., Kyoto, Japan) were cleaned with a piranha solution (7:3 mixture of concentrated sulfuric acid and 30% hydrogen peroxide). The glass plates were rinsed with water and 2-propanol, and then dried under a stream of dry nitrogen gas. A chromium layer (1 nm thick) and then a gold layer (49 nm thick) were deposited onto the glass plates using a thermal evaporator (V-KS200, Osaka Vacuum, Ltd, Osaka, Japan). The gold-coated glass plates were immersed in a solution of 1-hexadecanethiol (1 mM, in ethanol) at RT for 24 h to form a self-assembled monolayer (SAM) carrying methyl groups (CH_3_-SAM). The plates were washed with ethanol and dried in a stream of nitrogen gas.

### 2.4. Preparation of small unilamellar vesicles (SUVs)

SUVs were prepared by the conventional extrusion method.[21] Briefly, a solution of EggPC (in chloroform, 20 mg) was evaporated *in vacuo* for 12 h to form a lipid thin film*.* The resultant lipid film was mixed with PBS and vigorously stirred at 4°C for one day. Then, the lipid suspension was extruded through an 800 nm pore size membrane filter twice, a 220 nm filter twice, then a 100 nm filter for 10 times to prepare SUVs. The vesicle size was measured by dynamic light scattering (diameter: 105 ± 30 nm). The relatively large size distribution probably results from the high membrane fluidity of EggPC, as its transition temperature is rather low (–15 °C). The concentration of lipid in SUV suspension was determined by phospholipid C-test Wako.

### 2.5. Interaction between PEG-lipid and supported lipid membrane monitored by surface plasmon resonance (SPR)

A home-built SPR instrument was employed as reported previously.[22] A gold-coated glass plate with CH_3_-SAM was assembled with a flow cell and solutions were delivered to the flow cell at 3.0 ml min^–1^. All measurements were performed at 37 °C. The intensity of the reflected light was monitored during the flow of the liquid samples. To form supported lipid membrane, a suspension of SUV (100 μg ml^–1^) was flowed over the CH_3_-SAM substrate for 20 min, followed by wash with PBS. A solution of NH_2_-PEG-lipid (PEG-DMPE, PEG-DPPE, PEG-DSPE in PBS, 0.5 to 200 μg ml^–1^) was then applied to monitor interaction with the supported lipid membrane.

### 2.6. Fluorescence recovery after photobleaching (FRAP)

A glass coverslip (22 × 26 mm, Matsunami Glass Ind., Ltd, Osaka, Japan) was cleaned with a piranha solution, followed by rinse with deionized water and 2-propanol. To form a methyl-terminated monolayer on a glass substrate (CH_3_-glass), the glass plate was immersed into octadecyl triethoxysilane solution (5 (v/v) % in toluene) containing *n*-butyl amine (0.5 (v/v) %) for 1 h at RT followed by rinse with deionized water and ethanol.

To examine lateral fluidity of supported lipid membrane, a supported lipid membrane was prepared by SUVs containing L-α-palmitoyl-NBD dodecanoyl phosphatidylethanolamine as a fluorescent probe. A solution of EggPC (20 mg in 2 ml chloroform) was mixed with L-α-palmitoyl-NBD dodecanoyl phosphatidylethanolamine (0.2 mg) for the fluorescent probe at 1% molar ratio. SUVs containing the fluorophore were prepared as described in section 2.4. SUV suspension (100 μg ml^–1^ in PBS) was added onto CH_3_-glass surface to form a supported lipid membrane. After incubation for 1 h at 37 °C, the substrate surface was washed with PBS. The surface was then observed by a total internal reflection fluorescence microscope (ECLIPSE Ti-E, Nikon Co., Tokyo, Japan). NBD fluorophore was excited by light at 465–495 nm and fluorescence images were obtained through a bandpass filter (515–555 nm). In FRAP experiment, a circular area (diameter = 40 μm) of the microscope field was bleached for 5 s and fluorescence recovery of this area was observed. The lateral diffusion coefficient was calculated by the Soumpasis’s method.[23] For comparison, same experiment was performed using supported lipid bilayer membrane, which had been prepared by incubation of fluorescently labeled SUVs to a clean glass.

To study lateral fluidity of PEG-lipids on supported lipid membrane, a SUV solution without fluorescent probe was used. A CH_3_-glass surface was incubated with the SUV solution for 1 h, rinsed with PBS, and then incubated with a solution of FITC-PEG-lipid (200 μg ml^–1^ in PBS) for 1 h at 37 °C. After washing with PBS, the FRAP experiment was performed as described above.

### 2.7. Protein adsorption by SPR

BSA was chosen as a model protein to study protein adsorption to the supported lipid membrane using SPR. A supported lipid membrane was formed on a glass plate with CH_3_-SAM coating as described in section 2.5. A solution of MeO-PEG-DPPE (200 μg ml^–1^ in PBS) was then circulated to the lipid membrane for 1 h. Finally, a solution of BSA (5 mg ml^–1^ in PBS) was circulated for 30 min followed by washing with PBS to examine protein adsorption on the surface. All experiments were performed at 37 °C. The adsorbed amount of protein on the surface was calculated as follows:[22]

(1) $$\text{Amount of adsorbed protein}(\text{ng}/{\text{cm}}^{2})=500\times \Delta \text{SPR signal (deg)}$$

## 3. Results

### 3.1. Formation of supported lipid membrane by the vesicle fusion method

**Figure 1.**
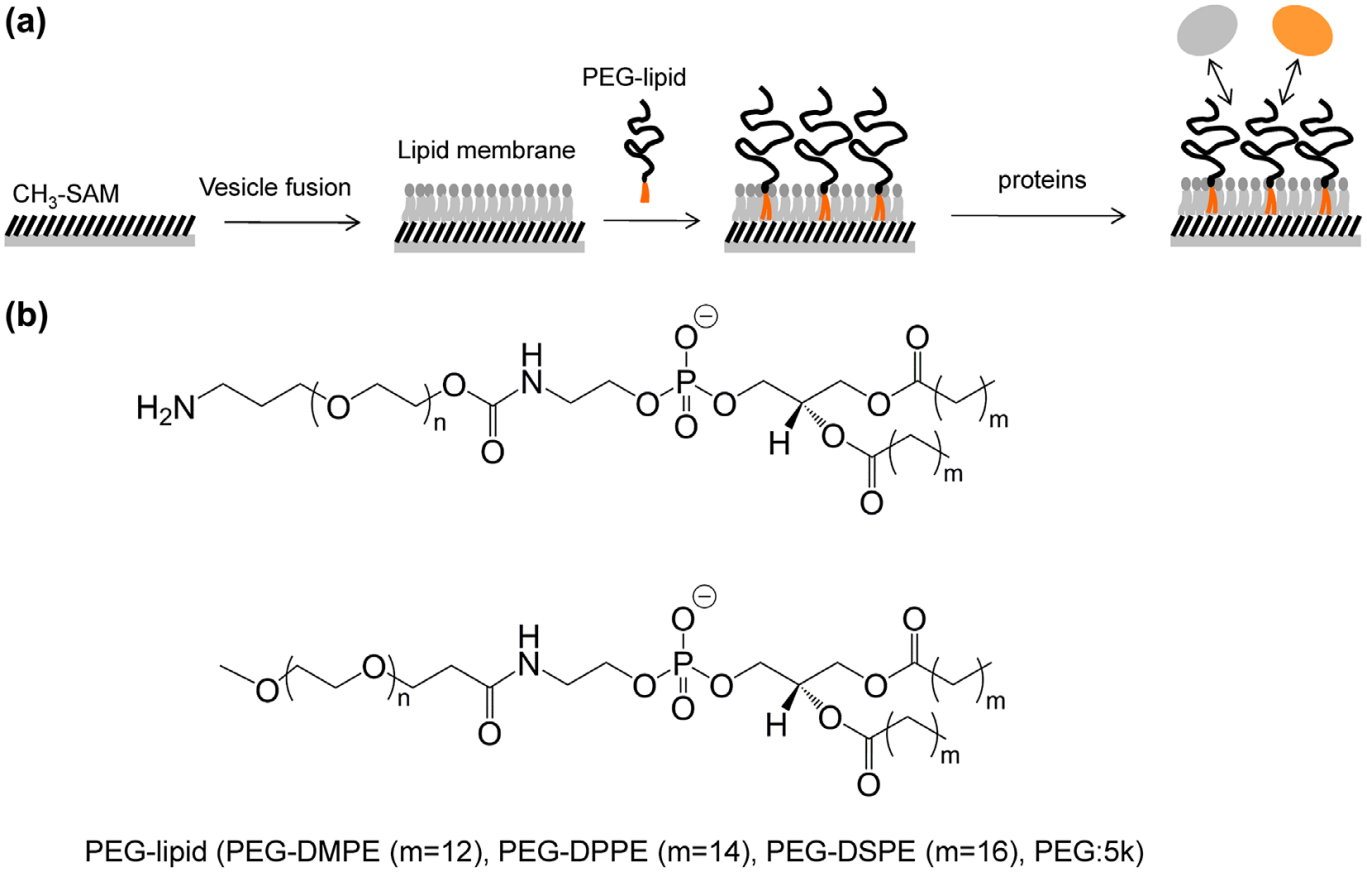
(a) Schematic illustration of PEG-lipid modified supported lipid membrane and interaction with serum proteins. (b) Chemical structures of PEG-lipids (PEG-DMPE (m = 12), PEG-DPPE (m = 14), PEG-DSPE (m = 16)).

**Figure 2.**
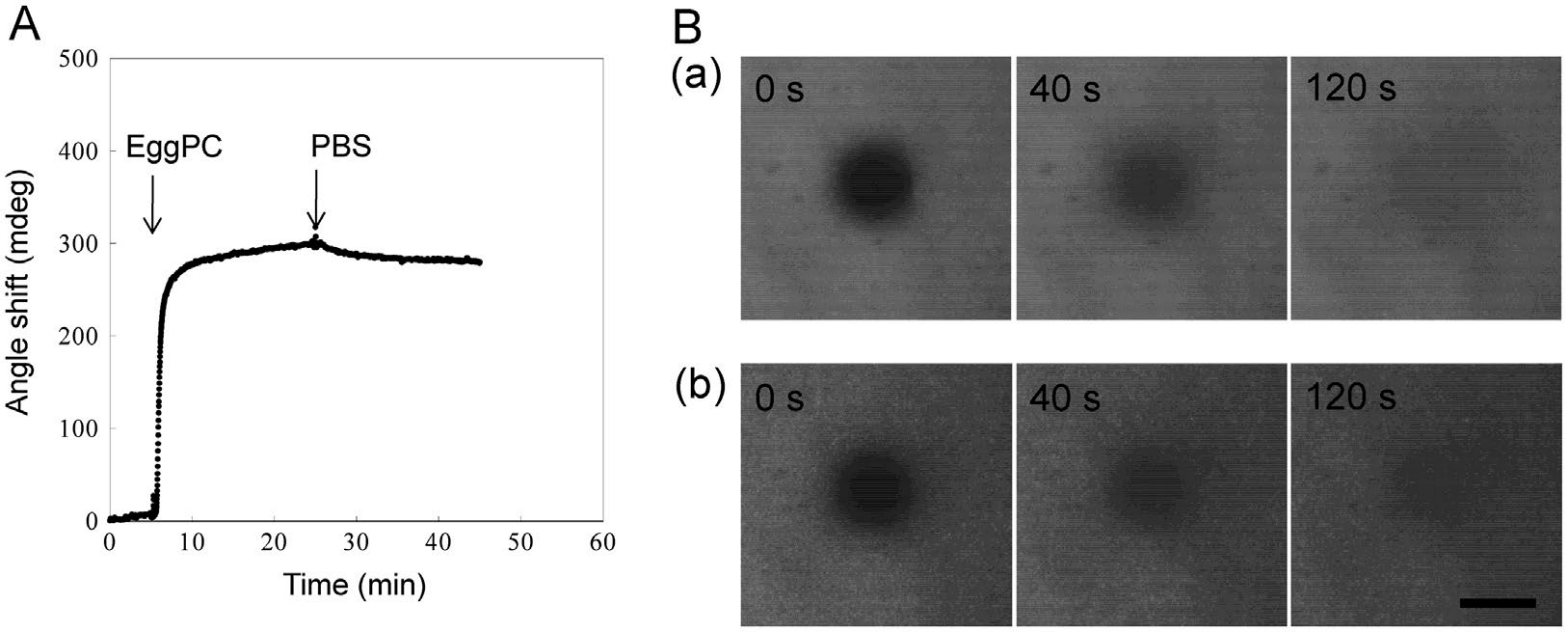
(A) An SPR sensorgram during exposure to EggPC SUV onto a CH_3_-SAM coated surface to form a supported lipid membrane. (B) Fluorescence images after photobleaching as a function of time for (a) supported lipid membrane formed on a hydrophobic CH_3_-glass surface and (b) supported lipid bilayer membrane on a glass surface. The images were taken at 0, 40, and 120 s after photobleaching of central region for 5 s. Scale bar = 40 μm.

Insertion and dissociation phenomena of PEG-lipids on a supported lipid membrane was monitored by SPR. A supported lipid membrane was formed on a CH_3_-SAM surface. Protein adsorption onto a supported lipid membrane modified with PEG-lipid was also examined (Figure 1). The vesicle fusion method using SUVs was used to form supported lipid membrane on a substrate.[24–26] In our study, vesicle fusion leads to the formation of a lipid monolayer on the hydrophobic CH_3_-SAM surface. We used a lipid monolayer on the hydrophobic surface since it is well established and suitable for preparation of lipid layer on gold. First, we monitored behavior of SUV on a CH_3_-SAM surface (Figure 2(A)). A large increase of the SPR signal was clearly observed after flowing of a SUV suspension, and indicates the physical adsorption of SUVs onto the surface. It is reported that SUVs attached to hydrophobic CH_3_-SAM surfaces fuse to the surface and then phospholipid molecules are spontaneously assembled to form a supported lipid membrane.[24–26] The lipid layer thickness calculated from the SPR signal was about 1.5 nm with WINSPALL software (available from W. Knoll lab, MPI, Mainz, Germany). The refractive index of lipid layer is supposed to 1.45. The calculated thickness is similar to the expected thickness of the single lipid monolayer although results include some uncertainty of lipid layer thickness based on composition of EggPC, which consists of phosphocholines carrying different acyl chains. This result suggests that lipid monolayer is formed on the CH_3_-SAM surface by the vesicle fusion method.

To further examine the formation of lipid membrane on CH_3_-SAM, we carried out the FRAP experiment, which is well-known method to examine lateral fluidity of molecules of interest. In this experiment, a CH_3_-surface on a glass cover slip was exposed to EggPC vesicles containing 1 mol.% L-α-palmitoyl-NBD dodecanoyl phosphatidylethanolamine to form fluorescently labeled supported lipid membrane (Figure 2(B) (part a)). After photobleaching of a circular area, fluorescence images were observed at intervals to measure changes in the fluorescence intensity of the photobleached area. The fluorescence was completely recovered in a few minutes, indicating the fluidity of the supported lipid membranes. The lateral diffusion coefficient was estimated to be 1.61 × 10^−8^ cm^2^ s^–1^. We also performed FRAP experiments for the supported lipid bilayer membrane, which was formed by a vesicle fusion on a hydrophilic glass surface (Figure 2(B) (part b)).[27,28] The lateral diffusion coefficient of the bilayer membrane on the glass was calculated to be 1.55 × 10^−8^ cm^2^ s^–1^ and was similar to that obtained for the monolayer membrane. This result indicates that the supported lipid monolayer membrane has almost the same property as the supported bilayer membrane and is acceptable as a model surface. Taken together with SPR results, the lipid monolayer was successfully formed on CH_3_–SAM surface. Therefore, the supported lipid membrane was used for further evaluations.

### 3.2. Interaction between PEG-lipid and supported lipid bilayer monitored by SPR

**Figure 3.**
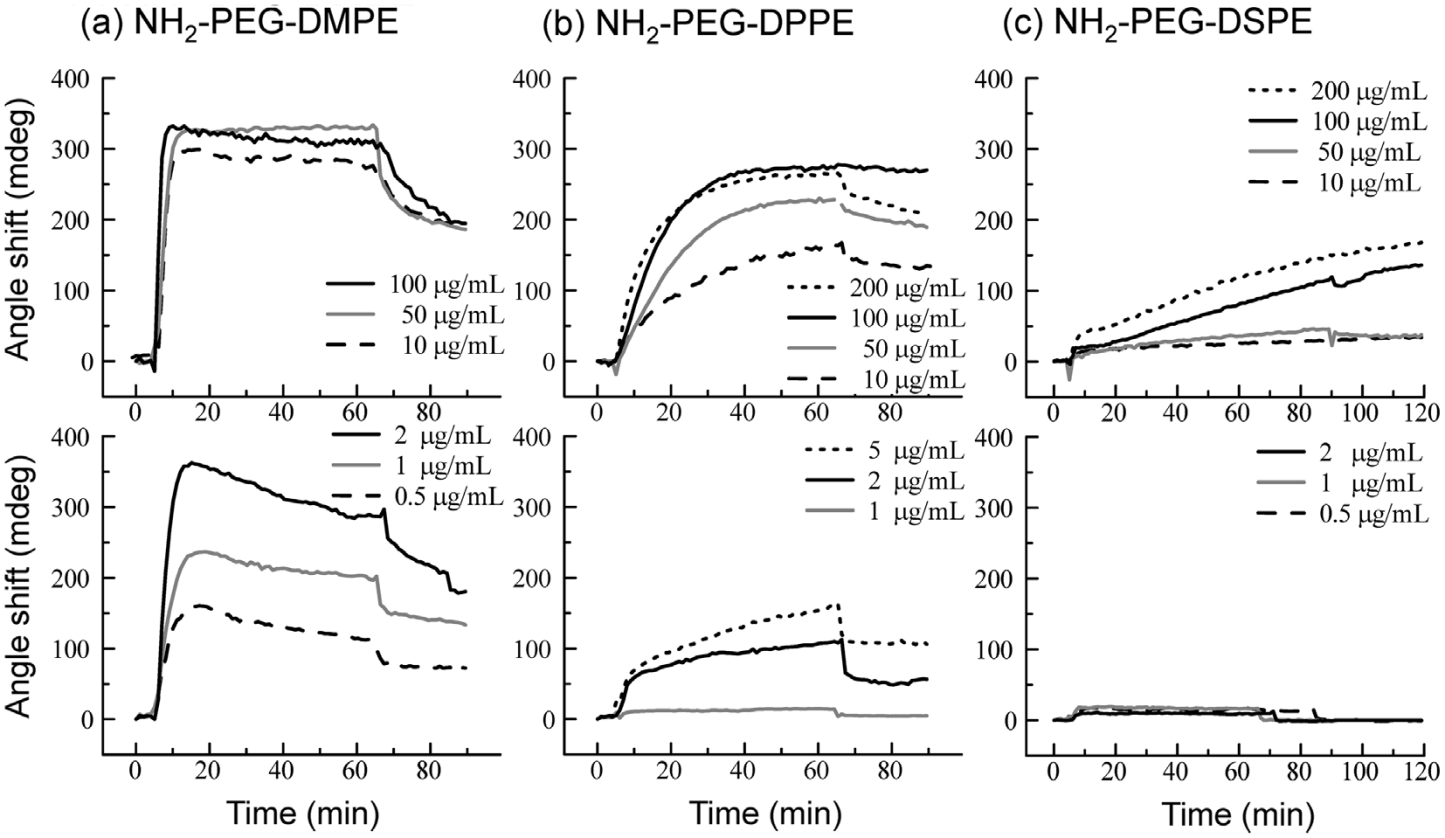
SPR sensorgrams when NH_2_-PEG-lipids ((a) NH_2_-PEG-DMPE, (b) NH_2_-PEG-DPPE, (c) NH_2_-PEG-DSPE) were applied onto the supported lipid membrane. The SPR sensorgrams at concentrations of NH_2_-PEG-lipids above 10 μg ml^–1^ are shown in the upper panels while those at concentrations below 5 μg ml^–1^ PEG-lipids are shown in the lower panels.

Behavior of PEG-lipids on the supported lipid membrane was studied by SPR (Figure 3). We used three kinds of NH_2_-PEG-lipids: PEG-DMPE, PEG-DPPE and PEG-DSPE with 5 k PEG, which differed in the length of the acyl chains (Figure 1(b)). When a PEG-lipid solution was applied onto a supported lipid membrane, incorporation into the membrane was observed for all PEG-lipids. For PEG-DMPE, the amount of incorporation was up to around 300 mdeg, and was saturated at above 2 μg ml^–1^, indicating that the lipid membrane was fully occupied with PEG-DMPE. During washing with PBS, dissociation of PEG-DMPE was clearly observed (Figure 3(a)). For PEG-DPPE, the amount of incorporation depended on the concentration of PEG-DPPE from 1 to 200 μg ml^–1^, reaching a plateau at around 300 mdeg as observed for PEG-DMPE. During washing with PBS, PEG-DPPE dissociated more slowly than PEG-DMPE (Figure 3(b)). In both cases of PEG-DMPE and PEG-DPPE, PEG densities on the supported lipid membrane can be estimated to be ~0.1 ng cm^–2^ from the signal after washing with PBS (refractive indices of PEG-lipids were supposed to 1.45). For PEG-DSPE, the incorporation rate was lower than PEG-DPPE and PEG-DMPE (Figure 3(c)). No incorporation of PEG-DSPE was observed during observation up to 2 μg ml^–1^ whereas the incorporation of PEG-DPPE and PEG-DMPE was observed at the same concentrations. Although the amount of incorporation increased with an increase of the concentration of PEG-DSPE ranging from 10 to 200 μg ml^–1^, the incorporation did not reach a plateau at these concentrations. No dissociation from the membrane was observed for PEG-DSPE during washing with PBS. Thus, a more hydrophobic acyl chain of PEG-lipid reduced both incorporation and dissociation of PEG-lipids on lipid membrane. We, therefore, concluded that hydrophobic interactions play an important role on the behavior of PEG-lipids on the lipid membrane.

### 3.3. Lateral mobility of PEG-lipid on supported lipid membrane

**Figure 4.**
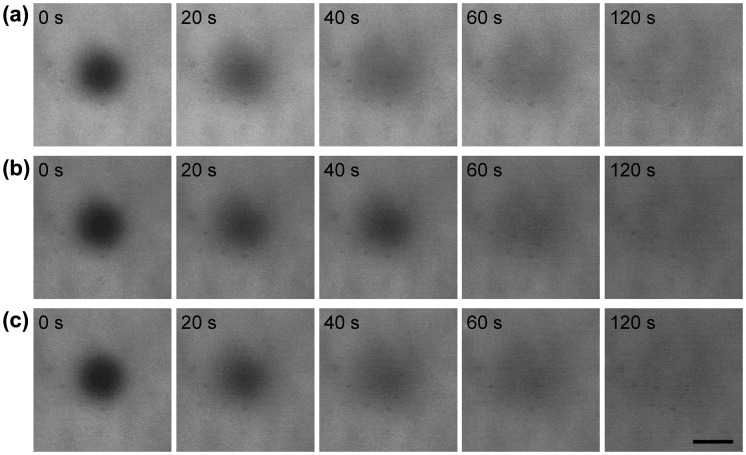
Fluorescence images after photobleaching as a function of time for FITC-PEG-lipids ((a) FITC-PEG-DMPE, (b) FITC-PEG-DPPE, and (c) FITC-PEG-DSPE) on a supported lipid membrane formed on CH_3_-glass. The images were taken after photobleaching of central region for 5 s. Scale bar = 40 μm.

The FRAP experiment was carried out to examine the lateral mobility of PEG-lipids on supported lipid membrane (Figure 4). We used three kinds of FITC-labeled PEG-lipids where FITC was conjugated at the terminal of a PEG chain. Before photobleaching, uniformly distributed fluorescence images were observed for all PEG-lipid-modified surfaces, indicating that the lipid membrane was uniformly modified with PEG-lipids. After photobleaching of a circular area, the fluorescence of PEG-lipids was completely recovered in a few minutes, indicating high lateral diffusion of PEG-lipids on the supported lipid membranes. The lateral diffusion coefficient was calculated to be 2.7 ± 0.2, 2.7 ± 0.7, and 2.9 ± 0.7 × 10^−8^ cm^2^ s^–1^, for PEG-DMPE, PEG-DPPE and PEG-DSPE, respectively. The lateral diffusion was not influenced by the hydrophobicity of PEG-lipids.

### 3.4. Interaction between BSA and PEG-lipid-modified supported lipid membrane

**Figure 5.**
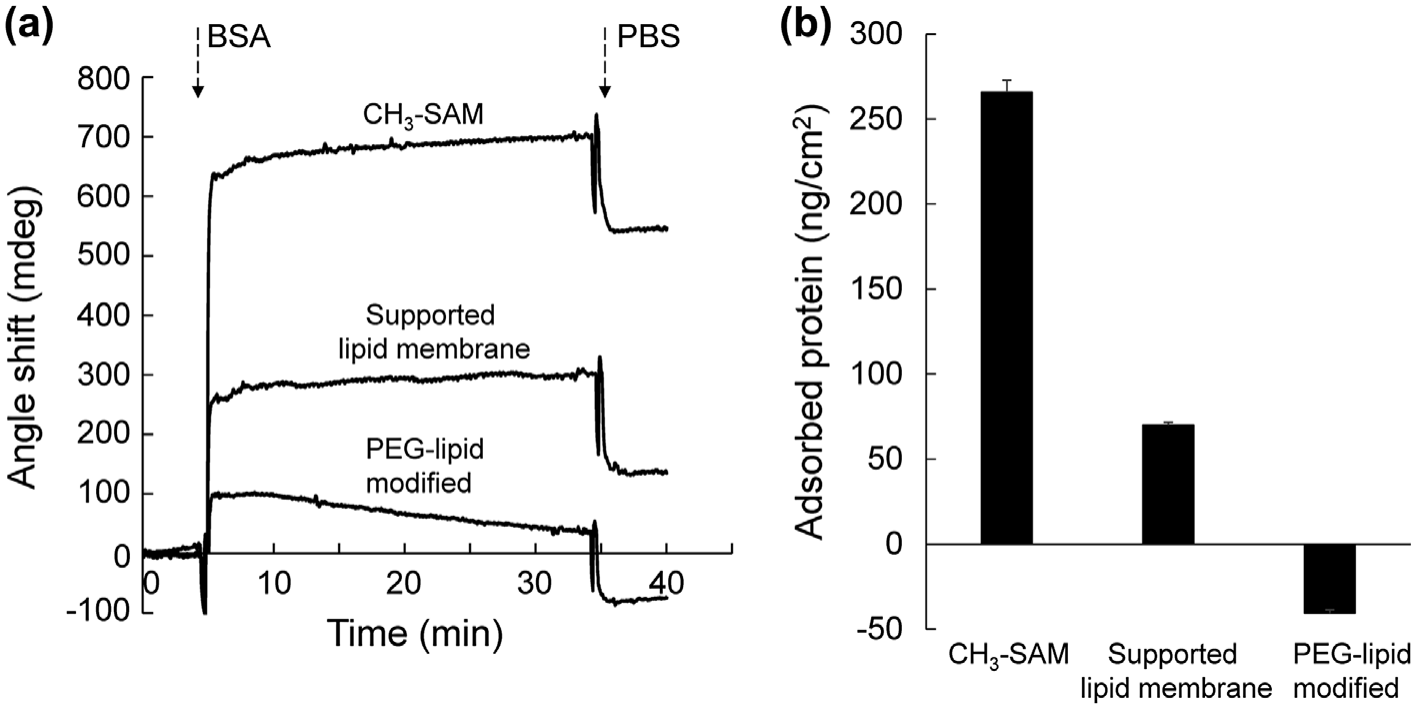
Influence of PEG-lipid modification on BSA adsorption. (a) SPR sensorgrams during exposure of BSA solution to CH_3_-SAM surface, supported lipid membrane, and MeO-PEG-DPPE-modified lipid membrane. (b) The adsorbed amount of BSA on surfaces. Results are presented as means ± SD (*n* = 3).

We next studied the adsorption of BSA onto a supported lipid membrane modified with MeO-PEG-DPPE to evaluate whether PEG modification effectively suppresses protein adsorption onto lipid membrane. We used BSA as a model protein since it is the most abundant protein in serum and therefore interacts with PEG-modified surfaces upon exposure to biological fluids. When CH_3_-SAM surface and supported lipid membrane were exposed to BSA, adsorption was clearly observed (265 and 70 ng cm^–2^, respectively) (Figure 5). On the other hand, MeO-PEG-DPPE modified lipid membrane exhibited negative value after exposure to a BSA solution. This negative value is attributed to the desorption of MeO-PEG-DPPE from lipid membrane since flowing buffer not containing BSA showed similar negative value (data not shown). These results demonstrate that MeO-PEG-DPPE modified membrane effectively suppress BSA adsorption.

## 4. Discussion

Our group has used amphiphilic polymers such as PEG-lipids and PVA-alkyls for surface modification of living cells.[16,18,19] Since amphiphilic polymers tend to disappear from the cell surface with time after surface modification,[16,18] it is necessary to understand the dynamic behaviors of amphiphilic polymers. However, real-time monitoring of these polymers on a living cell surface is difficult because both biological reactions and physicochemical reactions take place simultaneously. Therefore, supported lipid membrane with a neutral charge was used as a model membrane of cell surfaces, and the physicochemical interaction of PEG-lipids with lipid membrane was analyzed by SPR measurement. We observed that PEG-lipids were uniformly anchored to the lipid membrane with high fluidity without clustering. Additionally, the incorporation and dissociation rate of PEG-lipids into the supported membrane strongly depended on the length of the acyl chains; longer acyl chain decreases the incorporation rate and the dissociation rate of the PEG-lipids. No dissociation of PEG-DSPE could be observed on the supported lipid membrane. Similar phenomena were observed for modification of living cells. However, the dissociation of three kinds of PEG-lipids from cell surfaces was actually observed over time.[16,18,20] PEG-DSPE disappeared from living cell surfaces by 24 h although PEG-DSPE remained stably attached on the supported lipid membrane. Since most of the PEG-lipids did not exhibit cellular uptake by endocytosis, the PEG-lipids were considered to directly dissociate from the cell surface.[18] Therefore, the disappearance of PEG-lipids might be due to a biological exclusion process except endocytosis. Further study is needed to clarify exclusion pathways and to improve the retention time of amphiphilic polymers on the cell membrane.

FRAP experiments showed that PEG-lipids inserted into the lipid membrane diffused laterally on the membrane, indicating that PEG on the membrane was in a dynamic state. Thus, surfaces modified with PEG-lipid were totally different from surfaces covalently modified with polymers. Covalently attached polymers are usually in a static state, although polymer chains have some local mobility. While this static polymer-modified surfaces seem to be stable, that would not extend our understanding of cell surface modification with polymers because living cell membranes are in a dynamic state. Therefore, model cell membrane used in this study is useful to study dynamic behaviors of PEG-lipid and protein interactions. Modification of lipid membranes with PEG-lipids prevented the non-specific adsorption of BSA whereas lipid membrane alone exhibited protein adsorption, indicating the efficacy of PEG modification (Figure 5). Previously, we examined albumin adsorption to PEG-immobilized surface, which was prepared by conjugation of MeO-PEG-NHS to amine-terminated SAM. The amount of adsorbed albumin determined by SPR was ~ 40 ng cm^–2^ [29] and seems to be larger than that on lipid membrane modified with PEG-DPPE (in this study). These results suggest that PEG-lipids in a dynamic state effectively prevent protein adsorption. Further study is required to examine the effect of dynamic state on protein adsorption by considering characterization of PEG surfaces such as PEG density. In addition, adsorption of proteins is known to induce biological responses such as activation of the coagulation and the complement system,[17,30] which lead to loss of transplanted cells and liposomal drugs. PEG-modified supported lipid membrane would provide simple model to study these biological responses.

## 5. Conclusions

Supported lipid membranes can be modified with PEG-lipids through hydrophobic interaction. PEG-lipids are uniformly distributed on the lipid membrane and have high lateral fluidity. The incorporation and dissociation rate of PEG-lipids into a supported lipid membrane strongly depends on length of acyl chains. Insertion of PEG-lipids into lipid membranes prevents adsorption of albumin.

## Disclosure statement

No potential conflict of interest was reported by the authors.
